# Ultrasound-Assisted Anthocyanins Extraction from Pigmented Corn: Optimization Using Response Surface Methodology

**DOI:** 10.3390/mps6040069

**Published:** 2023-07-30

**Authors:** Annisa Nurkhasanah, Titouan Fardad, Ceferino Carrera, Widiastuti Setyaningsih, Miguel Palma

**Affiliations:** 1 Department of Food and Agricultural Product Technology, Faculty of Agricultural Technology, Gadjah Mada University, Jalan Flora, Bulaksumur, Depok, Sleman, Yogyakarta 55281, Indonesia; annisa.nurkhasanah@mail.ugm.ac.id; 2 Department of Physical Measurements, Institute of Technology of Lannion, CEDEX, 22302 Lannion, France; titouanfardad@gmail.com; 3 Department of Analytical Chemistry, Faculty of Sciences, Instituto de Investigación Vitivinícola y Agroalimentaria (IVAGRO), Agrifood Campus of International Excellence (CeiA3), University of Cadiz, Puerto Real, 11510 Cadiz, Spain; ceferino.carrera@uca.es (C.C.); miguel.palma@uca.es (M.P.)

**Keywords:** anthocyanins, Box–Behnken design, optimization, purple corn, ultrasound-assisted extraction

## Abstract

This study aimed to determine the optimal UAE conditions for extracting anthocyanins from pigmented corn using the Box–Behnken design (BBD). Six anthocyanins were identified in the samples and were used as response variables to evaluate the effects of the following working variables: extraction solvent pH (2–7), temperature (10–70 °C), solvent composition (0–50% methanol in water), and ultrasound power (20–80%). The extraction time (5–25 min) was evaluated for complete recovery. Response surface methodology suggested optimal conditions, specifically 36% methanol in water with pH 7 at 70 °C using 73% ultrasound power for 10 min. The method was validated with a high level of accuracy (>90% of recovery) and high precision (CV < 5% for both repeatability and intermediate precision). Finally, the proposed analytical extraction method was successfully applied to determine anthocyanins that covered a wide concentration range (36.47–551.92 mg kg^−1^) in several pigmented corn samples revealing potential varieties providing more health benefits.

## 1. Introduction

Pigmented corn is recognized by orange, red, purple, and blue kernels [1,2]. In Indonesia, varieties of purple and rainbow kernels are commonly available. Therefore, anthocyanins are traditionally considered natural food coloring agents [3,4] and have been used in Indonesia to reduce the use of artificial colorants. The varied colors of pigmented corn kernels are defined by anthocyanins, which provide essential health benefits such as anti-oxidant, anti-diabetic, anti-cancer, anti-inflammatory, and anti-obesity [5,6].

Several studies have reported high levels of anthocyanins in pigmented corn. Anthocyanin content in pigmented corn from Australia ranges from 2.2 to 4.4 g kg^−1^ [7]. The highest reported anthocyanin content in whole fresh purple corn (16.4 g kg^−1^) was higher than that in blueberries (3.9 g kg^−1^) [8]. Because it is a prominent source of anthocyanins, a reliable method for identifying and quantifying anthocyanins in various pigmented corn is necessary. 

Solid–liquid maceration is the foremost extraction treatment for anthocyanins [9]. In some cases, maceration to extract anthocyanins from purple corn requires a long extraction time of up to 3 h, thereby promoting the degradation of anthocyanins [10,11,12,13]. Therefore, advanced technology to accelerate extraction is continuously being developed to increase efficiency [14].

Ultrasound-assisted extraction (UAE) is widely used because it is not limited by the type of solvent, has low solvent consumption, and has a fast extraction time, thereby preventing component damage [15,16,17]. UAE provides higher anthocyanin recovery than microwave-assisted extraction and maceration [12,18,19,20,21] by performing extraction in a shorter time to avoid the breakdown of anthocyanin during the process [14].

The principle of analyte extraction from a solid matrix into a solvent is related to the cavitation effect produced by ultrasound. The type of solvent and composition of the mixture are essential factors because they are necessary for the cavitation effect on the surface of the solid sample. Another critical factor is the solubility of the target compound in the solvent medium. Anthocyanins are highly soluble in water and polar organic solvents, whereas their glycoside forms are very soluble in pure water [3,22]. Therefore, a mixture of polar organic solvents and water is suitable for extracting anthocyanins [23,24]. Methanol was chosen as the extraction solvent because of its higher effectiveness in anthocyanin extraction than ethanol [3,18,25]. A mixture of methanol and water was used to extract anthocyanins from corn [7,26,27].

Factors associated with the chemical properties of the solvent, such as temperature and pH, have been reported to affect extraction recovery [11,28] significantly. Anthocyanins in pigmented corn include cyanidin, pelargonidin, and peonidin [7,27,29], which are generally stable in acidic solutions, but the latter compound is also durable in high-pH solutions [3]. Other factors related to mass transfer effects, such as ultrasound power, pulse duty cycle, and solid: liquid ratio, also considerably affect extraction efficiency [28,30,31,32].

As many factors must be optimized, a Box–Behnken design (BBD) in conjunction with response surface methodology can help determine the condition providing the highest recovery of anthocyanins from pigmented corn. Therefore, this study aimed to optimize and validate the UAE method for recovering anthocyanins from pigmented corn. The proposed analytical method was subsequently applied to determine anthocyanins in various pigmented corns.

## 2. Materials and Methods

### 2.1. Chemicals and Reagents

HPLC-grade methanol was obtained from Fisher Scientific (Loughborough, UK). The water was purified using a Milli-Q water purification system (Millipore, Bedford, MA, USA). The pH of the extraction solvent was adjusted using 0.1 N hydrochloric acid (HCl), 0.1 M sodium hydroxide (NaOH), and formic acid (Panreac, Barcelona, Spain). Analytical grade cyanidin 3-*O*-glucoside, pelargonidin-3-*O*-glucoside, and peonidin-3-*O*-glucoside were obtained from Sigma-Aldrich (St. Louis, MO, USA).

### 2.2. Samples

**Figure 1:**
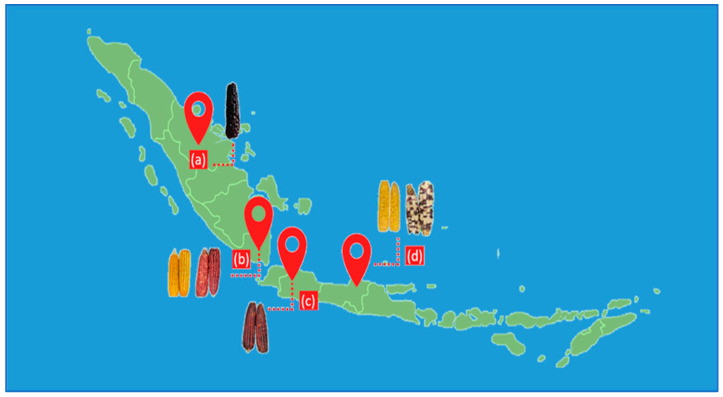
Corn samples collected from Indonesian Provinces: (a) Riau, (b) Lampung, (c) West Java, and (d) Central Java.

Six corn samples, including red, yellow, purple, and mixed-color kernels, were obtained from the local market in Indonesia (Figure 1). The samples were traditionally sun-dried by a farmer and stored at ambient temperature. The dried samples were then milled using a grinder (ML 130 Type SP-7406, JATA, Tudela, Spain) for 5 min with an on-off interval every 30 s and passed through a 1 mm screen mesh using a vibratory sieve shaker (AS 200, Retsch GmbH, Haan, Germany). The sample powder was homogenized and stored in a closed container until analysis. A mixture of samples in the same proportions was used to optimize the UAE experiments.

### 2.3. Ultrasound-Assisted Extraction (UAE)

Anthocyanin extraction from pigmented corn was performed based on Gonzales et al. [33], with slight modifications. UAE utilized a Sonopuls HD 4200 Ultrasonic Homogenizer (20 Hz, 200 W, Bandelin Electronic GmbH & Co. KG, Heinrichstrabe, Berlin, Germany) with a titanium probe TS 104 (diameter 4.5 mm). The extraction temperature was controlled using a water bath (Frigiterm-10, J.P. Selecta, Barcelona, Spain). The extraction for every 1 g sample employed 25 mL of solvent with a certain percentage of methanol in water at the defined pH based on the experimental design. The pH of the extraction solvent was adjusted using HCl 0.1 N and NaOH 0.1 M and measured using a Crison GLP22 pH meter (Barcelona, Spain). Extraction was carried out according to the experimental design for 5 min using 0.5 s^−1^ of a pulse duty cycle. Subsequently, the supernatant was separated from the solid material using a centrifuge (Centrofriger-BLT 230V, Selecta, Barcelona, Spain) at 4000 rpm for 10 min. The supernatant was then placed into 25 mL volumetric flasks to adjust the volume and pH (2). A 0.22 µm nylon syringe filter (Membrane Solutions, Dallas, TX, USA) was used to remove impurities before injecting the extract into the UHPLC-UV-Vis system.

### 2.4. Determination of the Anthocyanins by UHPLC-UV-Vis

Anthocyanin content was determined using UHPLC-UV-Vis based on Gonzales et al. [33]. An Elite UHPLC LaChrom System (Hitachi, Tokyo, Japan) equipped with an L-2200U autosampler, an L2300 column oven, and two L-2160U pumps was used for the chromatographic analyses. Separations were performed on a reverse-phase C18 column (2.1 × 50 mm and 2.6 µm particle size; Phenomenex, Kinetex, CoreShell Technology, Torrance, CA, USA). The injection volume was 15 µL. Elution was performed using mobile phases A (water with 5% formic acid) and B (pure methanol). Gradient separation was performed as follows: 2% B, 0.00 min; 2% B, 1.50 min; 15% B, 3.30 min; 15% B, 4.80 min; 35% B, 5.40 min; and 100% B, 6 min. The flow rate was 0.7 mL min^−1^. The column temperature was set to 50 °C. The system was coupled to a UV-Vis detector (L-2420U) and set at 520 nm for anthocyanin quantification. Assuming that the absorbance values of the various anthocyanins were similar and considering their molecular weights, the total anthocyanin content was calculated by summing the detected anthocyanins. The sum of anthocyanin-detected areas was measured and normalized to the experimental design response. A calibration curve prepared based on cyanidin 3-glucoside was used to quantify anthocyanins because more than 70% of the anthocyanins in pigmented corn are cyanidin-based compounds [7]. The analyses were performed in triplicate, and the results were expressed as mg of cyanidin 3-glucoside equivalents (CGE) kg^−1^ of dried corn kernel.

### 2.5. Identification of Anthocyanins Using UHPLC-PDA-QToF-MS

Anthocyanins in mixed samples were identified using a UHPLC system coupled to a photodiode array detector and a quadrupole time-of-flight mass spectrometer (UHPLC-PDA–QToF–MS) model Xevo G2 (Waters Corp., Milford, MA, USA). Identification was based on Gonzales et al. [33]. Separations were performed on a reverse-phase C18 column (100 × 2.1 mm and 1.7 µm particle size). Elution was performed using mobile phases A (water with 2% formic acid) and B (pure methanol). Gradient separation was performed as follows: 5% B, 0.00 min; 20% B, 3.30 min; 30% B, 3.86 min; 40% B, 5.05 min; 55% B, 5.35 min; 60% B, 5.64 min; 95% B, 5.94 min; and 95% B, 7.50 min, with a flow rate of 0.4 mL min^−1^. Each analysis was conducted within 12 min, including 4 min to restore initial conditions. The electrospray was operated in the positive ionization mode. The desolvation gas temperature was 500 °C, and the flow rate was 700 L h^−1^. The source temperature was 150 °C, and the capillary cone was set to 700 V. The cone voltage was set to 20 V with a gas flow of 10 L h^−1^. The trap collision energy was 4 eV. The full scan mode was used to identify anthocyanins in the 100–1200 *m*/*z* range.

**Figure 2:**
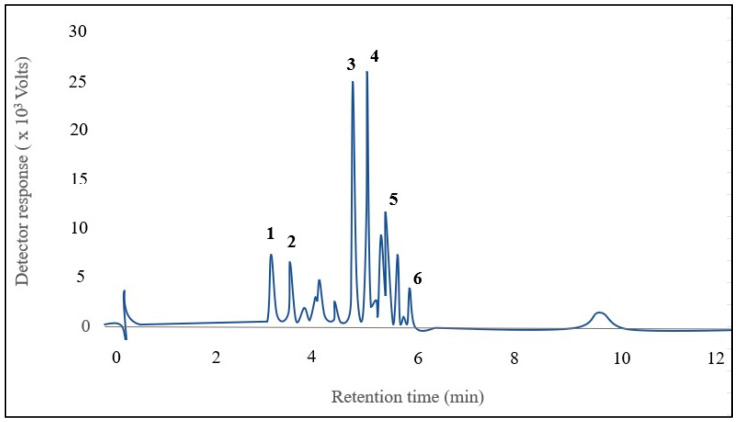
Chromatogram of 6 anthocyanins identified in the mixture of pigmented corns: 1. Cyanidin-3-glucoside, 2. Pelargonidin-3-glucoside, 3. Peonidin-3-glucoside, 4. Cyanidin-3-malonyl glucoside, 5. Pelargonidin-3-malonyl glucoside, 6. Peonidin-3-malonyl glucoside.

Six anthocyanins were identified in kernel corn samples (Figure 2). The major anthocyanins [M^+^] were identified as cyanidin-3-glucoside (*m*/*z* 449), pelargonidin-3-glucoside (*m*/*z* 493), peonidin-3-glucoside (*m*/*z* 463), cyanidin-3-malonyl glucoside (*m*/*z* 535), pelargonidin-3-malonyl glucoside (*m*/*z* 519), and peonidin-3-malonyl glucoside (*m*/*z* 549). The identified anthocyanins were the same as those described previously by Colombo et al. [1].

### 2.6. Experimental Design and Statistical Analysis

Box–Behnken design (BBD) with Response Surface Methodology was used to study factors that may affect the extraction efficiency of anthocyanins from pigmented corn, namely pH, temperature, solvent composition, and sonication power. The total area covered by the six identified anthocyanins was used as the target response. A BBD with four independent variables at three levels of factor values (−1, 0, and 1) was carried out. The independent variables and their levels are listed in Table 1. The overall design consists of 27 basic experimental units, as detailed in Table 2.

MINITAB (version X) (Minitab LLC, State College, PA, USA) generated the BBD and established the RSM model. An analysis of variance (ANOVA, *p =* 0.05) in conjunction with the Least Significant Difference (LSD, *p* = 0.05) test was used to determine the significance of the difference between the means.

### 2.7. Determination of the Optimal Extraction Time

The optimum extraction condition proposed by the RSM was used to evaluate the extraction time. The optimal extraction time was determined by assessing the level of anthocyanins at different extraction times (5, 10, 15, 20, and 25 min). The experiment was performed in triplicate using a randomized block design.

### 2.8. Method Validation

The analytical method was validated based on ICH Q2(R1) guidelines [34]. In addition to evaluating the analytical properties of the chromatographic procedure, the precision and accuracy assessments of the extraction method were also included in the method validation. The precision of the method was evaluated by performing repeatability (intra-day) and intermediate precision (inter-day). Repeatability was assessed by repeating nine analyses from a sample on the same day (*n* = 9), while intermediate precision was evaluated by performing three extractions on three consecutive days (*n* = 3 × 3). Precisions were expressed as the coefficient of variation (%CV). The trueness of the method was assessed by calculating the UAE recovery (%R), which was determined by comparing the total anthocyanin areas of the samples with and without spiking. The recovery was performed by adding the concentrated sample as a spike solution in the 25–40% range.

## 3. Results and Discussion

### 3.1. Performance of the Chromatographic Method

The total area of the detected anthocyanins was the response used for the optimization step, and the determination method for the compounds was validated for quality assurance. Hence, chromatographic analysis was validated by measuring the linearity, limit of detection (LOD), limit of quantification (LOQ), and precision of the method. A calibration curve (y=168.154x−28.642) was prepared using the cyanidin 3-glucoside standard (1−48 mg L^−1^) and measured at 520 nm. The resulting coefficient of determination (*R^2^*) was 0.9999, as described by Gonzales et al. [33]. The slope and standard deviation from regression were included in the calculation to define LOD (0.38 mg L^−1^) and LOQ (1.18 mg L^−1^). The molecular weights of the other anthocyanins (pelargonidin, peonidin, and malonyl derivatives) were used to calculate the total level of anthocyanins and were expressed as cyanidin glucoside equivalents (CGE). The precision of the peak area in the chromatographic results was evaluated by performing intra-day (CV, 1.52%) and inter-day (CV, 2.11%) injections on the sample. The resulting CV values were less than the acceptable level (2.7%) for analyte concentrations of 1–48 mg L^−1^, confirming the high precision of the method [35].

### 3.2. Effect of the UAE Operating Variables in the Recovery of Anthocyanins

**Figure 3:**
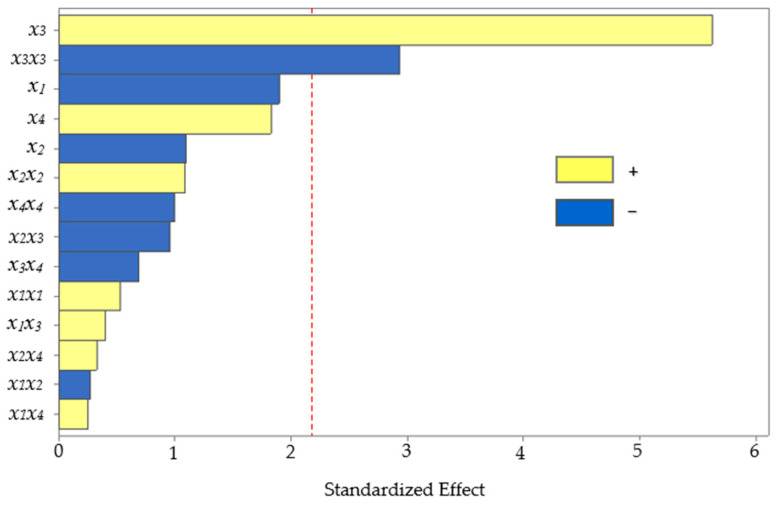
Pareto chart of the standardized effects from the UAE factors (*x*_1_, pH; *x*_2_, temperature; *x*_3_, solvent composition; and *x*_4_, power of sonication).

The BBD, consisting of 27 experiments, including three central points, was completed. Subsequently, the effect of the studied UAE factors on the level of anthocyanins extracted from corn kernels was assessed using analysis of variance (ANOVA). The statistical significance of each effect provided by the UAE factors was obtained by comparing the mean square error with the estimated experimental error. The standardized values (*p* = 0.05) in descending order of importance are plotted on a Pareto chart for the main, interaction, and quadratic effects (Figure 3).

The bars crossing the vertical line indicate the factors or combinations that significantly affect the response. The two effects had *p*-values below 0.05, marking a significant difference at the 95% confidence level. The main effect of solvent composition positively influenced (*p* < 0.001) the extraction, which means that the higher the amount of methanol in water, the higher the recovery of anthocyanins. The solvent composition has previously been reported as an influential variable for the extraction of anthocyanins from other similar matrices, such as purple corn cobs [4,36], purple corn flour [37], and red rice bran [38]. Hydroalcoholic mixtures are also more efficient than pure solvents for extracting moderately polar or amphiphilic molecules such as anthocyanins [39,40]. The polarity agreement between the analyte and the extraction solvent could increase the solubility. Improving the methanol in water as the extraction solvent provided higher recovery because the solubility of anthocyanins increased, facilitating the mass transfer rate.

However, the quadratic solvent composition showed a significant effect (*p* = 0.013) with an inverse relationship, which means that excess methanol in the solvent lowered the recovery. Excess methanol in the solvent would decrease the extraction yield because it creates a difference in polarity between the analyte and the extraction solvent, affecting the solubility.

### 3.3. Prediction Model Using Response Surface Methodology

**Figure 4:**
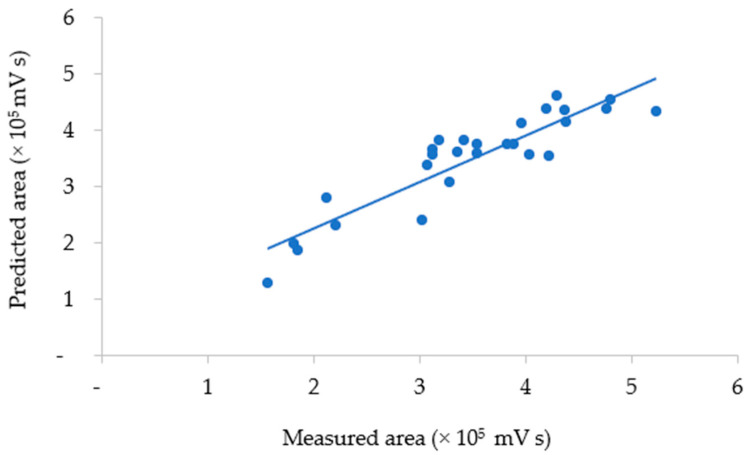
The measured and predicted areas of total anthocyanins in UAE extract.

The purpose of optimization using Response Surface Methodology (RSM) was to obtain the best combination of UAE factors to achieve the highest recovery results. A mathematical model of a second-order polynomial Equation (1) was established, considering the significant main and quadratic effects of the solvent composition on the level of extracted anthocyanins.

(1) $$y=0.7169+0.1831x_{3}-0.1431x_{3}x_{3}$$

where *y* is the total area of anthocyanin as a response, *x*_3_ is the solvent composition, and *x*_3_*x*_3_ is the quadratic solvent composition. The correlation between the measured and predicted values of total anthocyanins obtained using the model is plotted in Figure 4.

**Figure 5:**
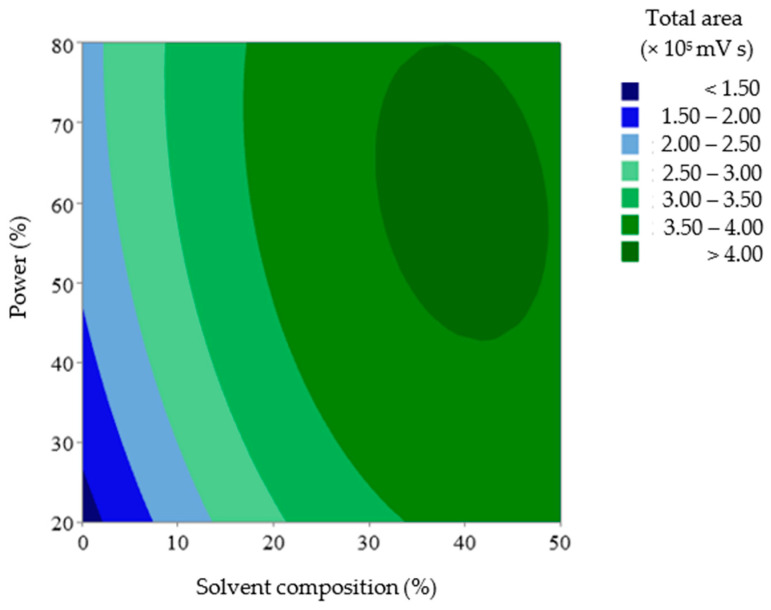
2D Surface plot diagram of the level of anthocyanins in UAE extracts by solvent composition and power.

The agreement between the measured and predicted total anthocyanin values was evaluated by the average relative prediction error (9.73%). The measured vs. predicted data values showed low variability (0.03–26.60%) around the mean value. A lack-of-fit test was also performed to determine whether the selected model was suitable for describing the measured data results. The resulting *p*-value for lack of fit (0.07) was non-significant, indicating that the model chosen satisfactorily represented the data at the 95% confidence level [30,31,39]. Hence, the equation model could describe the conditions of the UAE factors that defined the response with satisfactory predictions, as plotted in Figure 5.

The dark blue area represents the lowest relative area, whereas the dark green area represents the highest relative area. In this case, the region representing the highest recovery in the DOE was obtained when the solvent composition and ultrasound power approached their highest values in the studied range.

### 3.4. Optimization Conditions and Verification

The experimental design results suggested that the optimal extraction condition was set at +0.43 for solvent composition, while the remaining conditions were at a level of +1 for pH, +1 for temperature, and +0.78 for ultrasound power. Therefore, optimized extraction of anthocyanins from mixed pigmented corn samples using UAE could be achieved by applying 35.86% methanol in water at a pH of 7, a temperature of 70 °C, and a sonication power of 73.33%. The predicted anthocyanin area was 495.52 V s.

Three experiments were conducted to verify the optimum condition using 36% methanol in water with a pH of 7 at 70 °C and a sonication power of 73%. The resulting level of anthocyanins, indicated by the peak area, was 449.57 ± 3.44 V s, with a deviation of 9.27% from the prediction. These findings suggest that the Box–Behnken design successfully optimized anthocyanin extraction with RSM.

Muangrat et al. [4] conducted optimization research on extracting anthocyanin from purple corn cobs using UAE. The optimal conditions were similar to those determined in this study, namely the temperature (64 °C). However, the solvent composition (50% ethanol in water) and ultrasound amplitude (50 °C) differed from those observed in this study; both factors could be closely related to the various parts of corn.

### 3.5. Optimal Extraction Time

**Figure 6:**
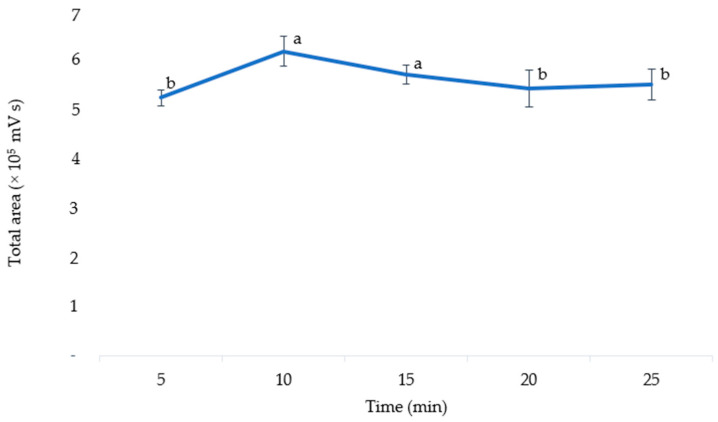
The total area of anthocyanins according to the different extraction times. The means with different letters are significantly different (*p* = 0.05).

The optimal extraction time was determined by varying the extraction time (5, 10, 15, 20, and 25 min). An ANOVA was used to analyze the results, which showed that the extraction time significantly affected the extraction efficiency (*p* = 0.018). As shown in Figure 6, when the extraction time was increased to 10 min, the level of anthocyanins in the extract also increased. However, this level decreased when the extraction time was increased to 20 min. A prolonged heating process may result in the degradation of thermolabile compounds [41]. Extracted anthocyanins are also easily oxidized by the environment if the extraction time is too long at high temperatures [42]. Thus, the extraction time of 10 min was chosen as the optimal extraction time for anthocyanins in pigmented corn. This value was much lower than the extraction times optimized in purple corn cobs with UAE (30 min) [4], MAE (19 min) [13], and in purple corn kernels with stirred maceration (30 min) [1].

### 3.6. Validation of the UAE Method

The accuracy of the method was assessed by measuring UAE recovery (%R). The anthocyanin extraction recovery was 92.81%. According to the AOAC guidelines (80–110%), the recovery results were within the accepted range, indicating that the optimal method has a high level of accuracy [35]. The precision of the method was evaluated by performing repeatability (intra-day) and intermediate precision (inter-day) tests. The results of the repeatability and intermediate precision tests were 4.62 and 4.61%, respectively. The acceptance limit for the precision of the analyte at a concentration of 1–10 mg L^−1^ was 11.0% [35]. The CV value obtained was less than the acceptance limit by the AOAC; thus, the proposed method can be validated as providing high-precision results.

### 3.7. Applying the Optimized Method to Different Pigmented Corn

The extraction method was applied to extract anthocyanins from pigmented corn to recover anthocyanins from various samples (Table 3). The studied pigmented corn contained anthocyanins in the 36.47–551.92 mg CGE kg^−1^ range. Purple corn had the highest anthocyanin content compared with red and white-purple corn. Purple corn has a high chroma and is positively correlated with anthocyanins [36].

Red corn from Lampung had a higher anthocyanin content than the red variety from West Java. This is because red corn from Lampung has thicker red corn bran than that from West Java. In addition, red corn from West Java has a thicker white endosperm than red corn from Lampung. Purple corn from other regions of Indonesia also contains different anthocyanins. For example, Manado has an anthocyanin content of 341 mg CGE L^−1^, and Malang has an anthocyanin content of 376 mg CGE L^−1^ [10]. The anthocyanin content in corn can vary depending on several factors, one of which is the growing conditions, such as growth location [43].

## 4. Conclusions

A new ultrasound-assisted extraction (UAE) method was successfully optimized using the Box–Behnken design to extract anthocyanins from pigmented corn. The optimal condition was achieved by applying a solvent pH of 7 at 70 °C employing 36% methanol in water and 73% sonication power for 10 min. The proposed optimal method was validated with high levels of accuracy and precision. The developed UAE method was successfully applied to determine the anthocyanin content of pigmented corn from several regions of Indonesia. Pigmented corn contains anthocyanins in the 36.47–551.92 mg CGE kg^−1^ range. Based on these results, it can be concluded that the newly developed UAE method in this study is fast yet reliable for determining anthocyanin content in pigmented corn matrices.

## Figures and Tables

**Table 1:** Selected independent variables and their levels.

Independent Variables	Levels		
	−1	0	+1
*x*_1_, pH	2	4.5	7
*x*_2_, Temperature (°C)	10	40	70
*x*_3_, Solvent composition (% methanol in water)	0	25	50
*x*_4_, Ultrasound power (%)	20	50	80

**Table 2:** A Box–Behnken design for four factors with the measured responses.

DOE	Independent Variables				Relative Measured Value to Maximum Responses * (%)
	*x*_1_	*x*_2_	*x*_3_	*x*_4_	
1	−1	0	−1	0	34.55
2	−1	0	+1	0	59.63
3	0	−1	−1	0	35.45
4	+1	+1	0	0	82.22
5	0	0	+1	+1	60.95
6	0	0	−1	−1	29.90
7	0	0	+1	−1	64.09
8	0	−1	0	−1	58.85
9	0	0	−1	+1	42.26
10	+1	0	0	+1	83.42
11	0	+1	0	+1	91.01
12	0	0	0	0	74.21
13	0	−1	+1	0	100.00
14	−1	−1	0	0	67.62
15	0	+1	+1	0	83.69
16	−1	0	0	+1	80.78
17	0	+1	0	−1	77.10
18	−1	+1	0	0	75.68
19	0	0	0	0	67.73
20	0	−1	0	+1	65.36
21	+1	−1	0	0	80.27
22	−1	0	0	−1	62.75
23	+1	0	+1	0	91.86
24	0	+1	−1	0	40.66
25	+1	0	0	−1	59.63
26	0	0	0	0	73.13
27	+1	0	−1	0	57.73

* Relative value to the total chromatographic area (520 nm) in the experimental design.

**Table 3:** Anthocyanin content of different pigmented corn matrices.

Location	Color	Picture	Anthocyanin Content (mg CGE kg^−1^)
Lampung	Red	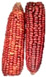	281.56 ± 31.29
West Java	Red	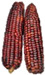	36.47 ± 6.65
Riau	Purple	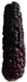	551.92 ± 14.02
Central Java	Purple and white	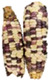	47.01 ± 4.32
Central Java	Yellow	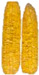	<LOQ
Lampung	Yellow	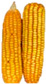	<LOQ

## Data Availability

The data presented in this study are available in this article.
